# A New Tool for *In Vivo* Study of Astrocyte Connexin 43 in Brain

**DOI:** 10.1038/s41598-019-54858-9

**Published:** 2019-12-04

**Authors:** Marine Droguerre, Tomokazu Tsurugizawa, Adeline Duchêne, Benjamin Portal, Bruno P. Guiard, Nicole Déglon, Nathalie Rouach, Michel Hamon, Franck Mouthon, Luisa Ciobanu, Mathieu Charvériat

**Affiliations:** 1 Theranexus, 60 Avenue Rockefeller, 69008 Lyon, France; 2 NeuroSpin, CEA, 91191 Gif-sur-Yvette, France; 3 Centre de Recherches sur la Cognition Animale (CRCA), Centre de Biologie Intégrative (CBI), Université de Toulouse, CNRS, UPS, 31330 Toulouse, France; 4 Laboratory of Neurotherapies and NeuroModulation, Neuroscience research Center (CRN), Lausanne University Hospital (CHUV) and University of Lausanne, 1011 Lausanne, Switzerland; 5 Laboratory of Neurotherapies and NeuroModulation, Department of Clinical Neuroscience (DNC), Lausanne University Hospital (CHUV) and University of Lausanne, 1011 Lausanne, Switzerland; 6 Laboratory of Neuroglial Interactions in Cerebral Physiopathology, Center for Interdisciplinary Research in Biology, Collège de France, CNRS UMR 7241, INSERM U1050, Labex Memolife, PSL Research University, Paris, 75005 France

**Keywords:** Magnetic resonance imaging, Glial biology

## Abstract

Astrocytes are glial cells organized in dynamic and structured networks in the brain. These plastic networks, involving key proteins such as connexin 43 (Cx43), are engaged in fine neuronal tuning and have recently been considered as emerging therapeutic targets in central nervous system disorders. We developed and validated a new application of the manganese-enhanced magnetic resonance imaging (MEMRI) technique allowing *in vivo* investigations of astrocyte-neuron interactions through quantification of brain Cx43 functional activity. The proof of concept has been achieved by quantification of MEMRI signals in brain after either local astrocyte-specific Cx43 knockdown with shRNA or systemic administration of Cx43 blockers. Unilateral hippocampal Cx43 genetical silencing was associated with an ipsilateral local increase of MEMRI signal. Furthermore, Cx43 blockers also enhanced MEMRI signal responses in hippocampus. Altogether, these data reveal the MEMRI technique as a tool for quantitative imaging of *in vivo* Cx43-dependent function in astrocytes under physiological and pathological conditions.

## Introduction

Data accumulated over the last decades mainly point out a role for glial cells in providing neurons with metabolic, structural and trophic support^1^. However, recent data have also shown that astrocytes are key non-neuronal cells involved in active signaling contributing to brain functions, as they notably regulate sleep-wake cycle, cognition^2^ and behavior^3^. At the cellular level, neuronal activity is tuned by astrocytes, in particular at tripartite synapses, that associate pre- and postsynaptic structures with an astroglial element^4^. Furthermore, astrocytes are organized in tightly regulated plastic networks^5^, through the transmembrane proteins connexins (Cx), especially connexin 43 (Cx43), which form hemichannels and gap junction channels^6^. A large body of evidence already shows that these networks are involved in neuronal modulation and play important roles in brain functions^3,7–9^.

To date, only few imaging markers of astrocytes such as positron emission tomography (PET) probes^10^, passive fluorescent dyes^11^ and genetically encoded calcium ions (Ca^2+^) indicators^12^, have been developed and successfully used. However, the *in vivo* usefulness of these probes is limited due to the requirement of their local intracerebral application^13^. *In vivo* brain imaging of the interactions between astrocytes and neurons is hence still challenging. We herein report data showing that manganese-enhanced magnetic resonance imaging (MEMRI) can capture those interactions. Indeed, previous findings using MEMRI suggest that: (i) manganese enters both neurons and astrocytes in brain^14^, (ii) astrocytes concentrate 80% of cerebral manganese ions (Mn^2+^)^15^, and (iii) Mn^2+^ shares chemical similarities with Ca^2+^, thus being able to enter neurons and astrocytes through Ca^2+^ channels and Na^+^/Ca^2+^ exchangers^14^. Accordingly, glial activation, notably during neuro-inflammatory processes^16^, neuronal tract tracing, and other specifically neuronal features could be studied using MEMRI. In our study, we developed and validated a new application of the MEMRI technique allowing the direct *in vivo* investigation of astrocyte-neuron interactions through quantification of hippocampus Cx43 functional activity. To this goal, we used *in vivo* administration of recombinant lentivirus targeting Cx43 expression and systemic treatments with known connexin modulators in mice.

## Materials and Methods

### Animals

Experiments were performed on 42 wild-type C57BL/6 male mice. Mice were housed 4–5 per cage under standard conditions (12/12 h light-dark cycle, light on at 7 am, 22 ± 1 °C ambient temperature, 60% relative humidity). Animal surgery and experimentations conducted in this study were approved by (i) the French Minister (Ministère de l’Education Nationale, de l’Enseignement Supérieur de la Recherche; France) and (ii) the local ethics committee (Comité d’Ethique en Expérimentation Animale, Commissariat à l’Energie Atomique et aux Energies Alternatives, Direction des Sciences du Vivant; Fontenay-aux-Roses, France) under reference: APAFIS#4082-2016021510499450v2). Experiments were conducted in strict accordance with the recommendations and guidelines of the European Union (Directive2010/63/EU) and the French National Committee (Décret 2013-118). All efforts were made to improve animal welfare and minimize animals suffering.

### Drugs and treatments

Mefloquine (MEF; Sigma-Aldrich), meclofenamic acid (MFA; Sigma-Aldrich) and flecainide acetate (FLE; Sigma-Aldrich) were freshly prepared in 0.9% NaCl with 2% DMSO and administered intraperitoneally (i.p.) at the same dose of 1 mg/kg in a volume of 5 mL/kg, 2.5 h before imaging. Control animals received the vehicle only under the very same conditions.

### Preparation and stereotaxic injection of lentiviral vectors

MOKOLA pseudotyped lentiviral vector (LV) encoding a shRNA directed against Cx43 (passenger-loop-guide strand: AACAGTCTGCCTTTCGCTGTA-TAGTGAAGCCACAGATGTA-TACAGCGAAAGGCAGACTGTT) or GFP (passenger-loop-guide strand: GCAAGCTGACCCTGAAGTTCAT-CTGTGAAGCCACAGATGGG-ATGAACTTCAGGGTCAGCTTGC) mRNA (control vector; shCTRL-LV) within astrocytes were generated as previously described^17,18^. For these recombinant viruses, the viral envelope ensures the specific tropism towards astrocytes. In addition, a detargeting strategy using miR9*T and miR124T was set up to eliminate any possible residual expression in neuronal cells^17–19^.

Mice were anesthetized with ketamine 75 mg/kg i.p. and xylazine 10 mg/kg i.p. LV were injected using a 34-gauge blunt-tip needle connected to a Hamilton syringe (Reno) with a polyethylene catheter. Dilutions of viral preparations in PBS containing 1% bovine serum albumin (BSA) were made so as to reach a final concentration of 100,000 ng p24 µL^−1^ in the suspensions for injections. Mice received unilateral injections of 1 µL LV suspension at a rate of 0.1 µL/min at three different sites into the hippocampus. ShCx43-LV was injected on one side (either right or left, randomly chosen) and shCTRL-LV was injected on the contralateral side (left or right, respectively) in each mouse. The stereotaxic coordinates for these three unilateral injections were respectively: site 1 = AP, −1.94; L, ± 2.00 and V, −1.50; site 2 = AP, −2.46; L, ± 2.50 and V, −2.60; site 3 = AP, −3.0; L, ± 2.7 and V, −3.5 (in mm from bregma^20^). At the end of each injection, the needle was left in place for 5 min before being slowly removed. At the end of the injection procedure, the skin was sutured with 4-0 silk thread (Mersilkä, Ethicon Inc.) and mice were allowed to recover under a heating light before returning in their home cage.

### MRI acquisitions

The MEMRI technique is based on the paramagnetic properties of Mn^2+^ which induce a marked reduction of the longitudinal T_1_ relaxation time during MRI. MRI signal acquisitions were performed on horizontal bore small animal scanners (Bruker BioSpin, Ettlingen, Germany) operating at 7 T and 11.7 T using a 4-channel mouse brain array coil and a two-channel mouse brain cryoprobe, respectively. The mice were anesthetized with 0.5–1.0% isoflurane in air throughout the experiments. The respiration rate was monitored and the body temperature was maintained at 37 °C using an MR-compatible, feedback-controlled air heating system (model 1025; SA Instruments, NY) or circulating hot water system. A capillary filled with 100 µM MnCl_2_ (in distilled water) was placed on the side of the head, inside the radiofrequency (RF) coil, and was used as reference for T_1_-weighted signal intensity normalization.

The T_1_-weighted images were acquired using a 3D rapid acquisition with relaxation enhancement (RARE) sequence: RARE factor 4, repetition time/echo time = 250 ms/6 ms, 4 averages. For T_1_ relaxation time measurements, we used a true Fast Imaging with Steady-state free Precession (trueFISP) sequence with the following acquisition parameters: repetition time/echo time = 3.66/1.83 ms, flip angle = 10, inversion time = 80 ms, number of inversion times = 60, 2 averages. The spatial resolutions and acquisition times for the two sequences on the two MRI systems are listed in Table 1.

**Table 1:** Data acquisition parameters for the two MRI devices (7 T and 11.7 T).

	Acquisition time (min)		Spatial resolution	
	7 T	11.7 T	7 T	11.7 T
RARE	80	47	0.1 × 0.1 × 0.1 mm^3^	0.1 × 0.1 × 0.1 mm^3^
FISP	60	70	0.15 × 0.15 × 0.15 mm^3^	0.1 × 0.1 × 0.1 mm^3^

RARE, 3D Rapid Acquisition with Relaxation Enhancement; FISP, Fast Imaging with Steady-state free Precession.

### Data processing

The position of the brain image was co-registered to a template using SPM8 software (Welcome Trust Center for Neuroimaging, UK). The template image was co-registered to mouse brain atlas^20^. T_1_-weighted signals were normalized using the signal intensity from the reference tube. T_1_ maps were calculated as described previously^21^. Eighteen regions of interest (ROIs) were defined using the mouse brain atlas. The averaged T_1_-weighted signals and T_1_ values were calculated using an in-house written Matlab program. To compensate for differences in coil sensitivity between left and right channels, we calculated the signal intensities in ROIs drawn in the left and right cerebral cortex, including the somatosensory cortex and insular cortex (ROI_cortex_left_ ROI_cortex_right_). The averaged T1-weighted signal intensity on the left side (SI_left_) was then corrected following the equation:

1 $$S{I}_{left\_corrected}=S{I}_{left}\frac{S{I}_{cortex\_right}}{S{I}_{cortex\_left}}.$$

### Immunohistochemistry

After MRI acquisitions, mice were deeply anesthetized and transcardially perfused with 4% paraformaldehyde in phosphate-buffered saline (PBS). Series of one in twelve 30 μm thick coronal sections were incubated in a solution of rabbit anti-GFAP (1:500, Agilent technologies) and mouse anti-Cx43 (1:250, BD transduction) overnight at 4 °C. Sections at the very injections sites and displaying signs of lesions were discarded. After several rinses in PBS containing 0.25% Triton-X100 (PBST), sections were incubated for 2 h at room temperature in a mixture of Alexa Fluor^®^ 488-conjugated highly cross-adsorbed donkey anti-rabbit and Alexa Fluor^®^ 555-conjugated donkey anti-mouse (all at 1:500; Life technologies, ThermoFisher) antibodies in PBST supplemented with 10% normal donkey serum and 0.1% BSA. Sections were finally rinsed extensively and mounted onto slides, coverslipped using Mowiol^®^ and stored at 4 °C.

On the other hand, coronal sections at hippocampal level from mice injected locally with shCx43-LV on one side and shCTRL-LV on the other side were labeled with the nucleic acid dye 4′6-diamidino-2-phenylindole (DAPI) to visualize all cell nuclei and verify that recombinant lentiviruses were devoid of any neurotoxic effect.

### Quantification of Cx43 deletion

Cx43-immunolabeled hippocampi were pictured for each injection site using an Olympus BX-51 microscope equipped with Mercator Software (Explora Nova, France). The dentate gyrus area was framed at x40 magnification lens for each injection site. Quantification of Cx43-labeled sections was conducted with the FIJI software (ImageJ 2, NIH). Dentate gyri of the three injection sites sections were defined as the region of interest and a square (800 × 800 px) was delineated on each injection site to measure the mean gray value. Value intensity of Cx43 gray label was normalized to the GFAP gray label value in the very same area. Results are presented as the mean ± SEM of the three injection sites gray value.

### Experimental design and statistical analyses

**Figure 1:**
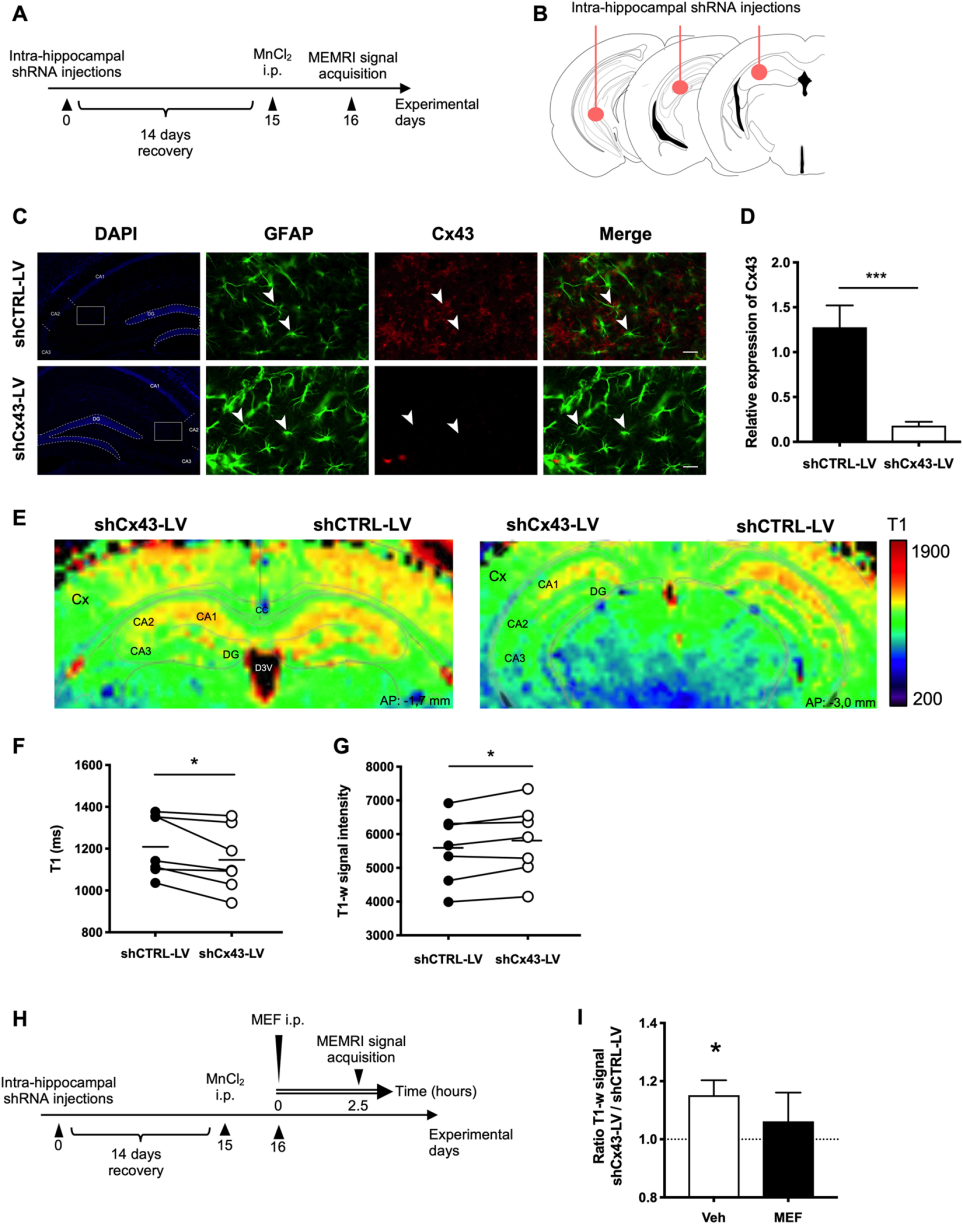
MEMRI signal responses to selective Cx43 knockdown expression in the mouse hippocampus. (**A**) Experimental timeline used for the experiments shown in E, G and Table 2. Mice received intra-hippocampal shRNA injections and were allowed to recover for 14 days. On day 15, MnCl_2_ (50 mg/kg) was delivered i.p. and MEMRI signal acquisition was performed 24 h later. (**B**) shRNA stereotaxic injection procedure. Mice received unilateral injections of LV suspension at three different sites into the hippocampus. ShCx43-LV was injected on one side and shCTRL-LV on the contralateral side (**C**) Representative fluorescence microscopy images of hippocampal shCTRL-LV- and shCx43-LV-injected sides showing DAPI (blue), GFAP+ cells (green) and Cx43 expression (red). Arrow heads point at typical double-labeled GFAP+/Cx43+ astrocytes (top; shCTRL-LV) or highlight the dramatic decrease in the hippocampal expression of Cx43 (bottom; shCx43-LV injected hippocampus). Scale bar: 20 µm. (**D**) Relative expression of Cx43 in the hippocampus on the shCTRL-LV versus the shCx43-LV-injected side. ***p < 0.001, Mann-Whitney test. Each bar is the mean ± SEM of 9 independent determinations. (**E**) Typical MEMRI signal responses maps (T1) at hippocampal level (AP = −1.7 mm and −3.0 mm^20^) in a mouse injected with shCx43-LV on one side and shCTRL-LV on the other side. Cx, Cortex; CA1, 2, 3, respective regions of cornu ammonis; DG, Dentate Gyrus; D3V, Dorsal 3rd ventricle. (**F**) Respective T_1_ (in ms) and (**G**) T_1_-weighted signal intensity values in the hippocampus on shCTRL-LV and shCx43-LV injected side. MR acquisitions were performed on a 11.7 T Bruker imaging system. Significant differences in both parameter values were observed between the two sides; *p < 0.05, paired *t* test. (**H**) Experimental timeline used for the experiments shown in I and Table 3. Mice received intra-hippocampal shRNA injections and were allowed to recover for 14 days. On day 15, mice received MnCl_2_ (50 mg/kg; i.p.) then MEF (1 mg/kg; i.p.) or vehicle 24 h later. MEMRI signal acquisition was performed 2.5 h after the MEF administration. (**I**) Data are presented as mean ± SEM of the ratio shCx43-LV/shCTRL-LV of MEMRI signal intensity in the hippocampus 2.5 h after i.p. administration of MEF or its vehicle. *p < 0.05, one sample *t* test compared to the hypothetical value 1, n = 6 in each group.

**Figure 2:**
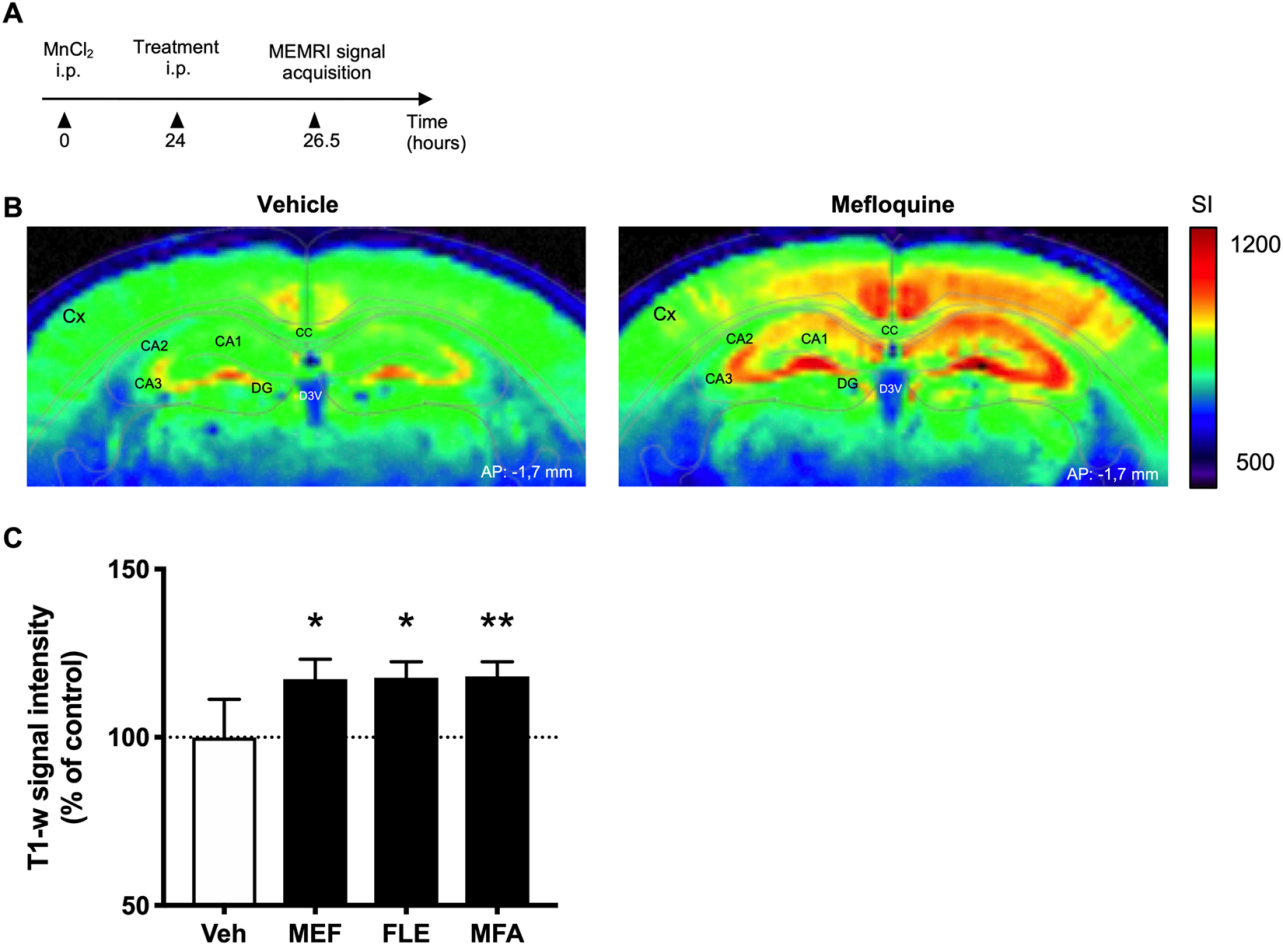
Effects of acute treatment with mefloquine (MEF), flecainide (FLE) or meclofenamic acid (MFA) versus vehicle (Veh) on MEMRI signal intensity in the mouse hippocampus. (**A**) Experimental timeline used for the experiments shown in B and C. Mice were injected with MnCl_2_ (50 mg/kg; i.p.) then received connexin blocker (MEF, FLE or MFA, all at 1 mg/kg i.p.) or vehicle 24 h later. MEMRI signal acquisition was performed 2.5 h after the latter injection. (**B**) T_1_-weighted signal intensity maps in the hippocampus (AP = −1.7 mm^20^) after vehicle or mefloquine administration. (**C**) Mice pretreated with MnCl_2_ were subjected to MEMRI 2.5 h after administration of each drug. MEMRI signal intensity in the hippocampus was compared to the respective value in vehicle-treated control mice. Data are presented as mean ± SEM. Cx, Cortex; CA1, 2, 3, respective regions of cornu ammonis; DG, Dentate Gyrus; D3V, Dorsal 3rd ventricle. *p < 0.05, **p < 0.01, n = 6 in each group.

Animals were divided into three sets of experiments as described in the experiment timelines (Figs. 1A,H and 2A): LV intra-hippocampal injections only (Set A, n = 7); LV intra-hippocampal injections and vehicle or MEF i.p. administration (Set B, n = 11); vehicle, MEF, MFA or FLE i.p. administration (Set C, n = 24). In set A, animals were injected with MnCl_2_ 50 mg/kg (in distilled water) i.p. two weeks after LV injections, and MRI acquisitions (see below) were performed 24 h later. In set B, mice were injected with LV and MnCl_2_ as in set A, then received MEF treatment 24 h after i.p. MnCl_2_ and 2.5 h before MRI acquisitions. In set C, mice received only MEF, MFA or FLE i.p. 24 h after i.p. MnCl_2_ and 2.5 h before MRI acquisitions.

Statistical analyses were performed using GraphPad Prism version 7.0c. Data were analyzed using Mann-Whitney non-parametric test, paired *t* test or one sample *t* test compared to the hypothetical value 1 or 100 as appropriate. In each experiment, a level of p < 0.05 was accepted as evidence for a statistically significant effect (*p < 0.05; **p < 0.01; ***p < 0.001). Changes in MEMRI signal in response to the Cx43 expression knock-down and/or drugs administration are expressed as means ± SEM of percent of control. All other data are expressed as mean ± SEM. For analysis of MEMRI signal responses to drugs administration, quantification was made in six brain areas selected on the basis of their different response patterns.

## Results

### Selective silencing of astrocyte Cx43 expression in the mouse hippocampus

Recombinant LV with a glial tropism and expressing shRNA against Cx43 (shCx43-LV) were stereotaxically injected into one hippocampus to generate mice with a unilateral specific inactivation of astroglial Cx43 in this region^19^. Recombinant LV expressing shRNA against GFP (shCTRL-LV) were injected into the contralateral hippocampus as control (Fig. 1B,C). DAPI labeling showed that the cell density in both hippocampi did not differ from each other and from that determined in control mice (not shown). The characteristic punctate Cx43-like immunoreactivity in shCTRL-LV injected hippocampus was shown to co-localize with GFAP-immunoreactive astrocyte (Fig. 1C). Quantification of immunostained brain sections confirmed that mice displayed a dramatic decrease in the hippocampal expression of Cx43 on their shCx43-LV-injected side relative to their shCTRL-LV-injected contralateral side (approx. −85%; p < 0.001; Mann-Whitney test; Fig. 1C,D).

### MEMRI signal responses to selective Cx43 knockdown expression in the mouse hippocampus

Mn^2+^ accumulation, because of its ability to shorten the longitudinal relaxation time (T_1_) of the proton, produces hyperintense regions on T_1_-weighted images. To assess the impact of Cx43 knockdown on Mn^2+^ accumulation_,_ we applied the MEMRI technique in shCx43-LV-injected mice which received MnCl_2_ via the i.p. route 24 h before the MEMRI acquisition. Figure 1E shows coronal T1 images of one representative mouse brain (−1.7 mm from bregma, left panel; −3.00 mm from bregma, right panel) 24 h after MnCl_2_ administration. A clear-cut decrease in MEMRI signal response appeared in the shCx43-LV injected hippocampus in comparison with the contralateral shCTRL-LV injected side (Fig. 1F). Quantification of images acquired with 7 T and 11.7 T MRI devices showed that T_1_ value for the hippocampus was significantly lower on the shCx43-LV injected side compared to the contralateral shCTRL-LV injected side (−1.42 ± 0.53% and −5.31 ± 1.65% with 7 T and 11.7 T MR imaging systems, respectively, p < 0.05; paired *t* test, Table 2). On the other hand, a significant increase of the T_1_-weighted signal intensity was observed on the shCx43-LV injected side in comparison with the contralateral shCTRL-LV injected side (+15.20 ± 5.16% and +3.91 ± 1.24%, with 7 T and 11.7 T MR imaging systems, respectively; p < 0.05; paired *t* test) (Fig. 1G).

**Table 2:** Effects of astroglial Cx43 expression knockdown in the hippocampus on MEMRI signal in various brain regions.

Region	Signal changes for shCx43-LV vs. shCTRL-LV (%)				Statistics			
	7 T		11.7 T		7 T		11.7 T	
	T_1_	T_1_-w	T_1_	T_1_-w	T_1_	T_1_-w	T_1_	T_1_-w
**Hip**	**−1**.**42 ± 0**.**53**	**15**.**2 ± 5**.**16**	**−5**.**31 ± 1**.**65**	**3**.**91 ± 1**.**24**	<**0**.**05**	<**0**.**05**	<**0**.**05**	<**0**.**05**
SC	0.40 ± 0.57	9.32 ± 6.58	−2.61 ± 4.91	3.12 ± 3.57	0.52	0.32	0.53	0.40
Cpu	−0.34 ± 0.49	10.3 ± 6.99	−3.08 ± 4.89	−0.69 ± 4.19	0.54	0.33	0.41	0.84
DRa	1.09 ± 1.56	8.67 ± 7.24	1.13 ± 5.09	0.67 ± 5.13	0.56	0.54	0.95	0.91
Hyp	0.59 ± 1.62	8.20 ± 7.65	−2.48 ± 4.58	3.59 ± 5.21	0.81	0.63	0.41	0.62
LC	0.52 ± 1.18	10.7 ± 6.20	−2.10 ± 3.68	−1.01 ± 4.13	0.66	0.22	0.46	0.86
LPB	−0.83 ± 1.14	13.7 ± 7.04	−6.56 ± 5.57	−2.00 ± 4.47	0.47	0.09	0.20	0.74
MC	0.58 ± 0.51	9.34 ± 6.57	−4.30 ± 3.81	0.47 ± 3.78	0.33	0.37	0.28	0.82
OB	−0.07 ± 0.39	9.61 ± 8.10	−5.88 ± 5.16	2.27 ± 5.27	0.76	0.60	0.26	0.86
SN	−0.85 ± 1.05	14.2 ± 6.74	−5.93 ± 5.96	0.54 ± 4.02	0.46	0.13	0.28	0.77
ThN	−0.23 ± 0.54	13.1 ± 6.72	−3.39 ± 2.92	−0.85 ± 4.40	0.68	0.21	0.23	0.86
OC	−0.42 ± 1.12	9.85 ± 7.68	−3.98 ± 5.06	0.75 ± 4.45	0.72	0.45	0.39	0.96
PrL	0.93 ± 1.87	7.59 ± 9.92	−2.36 ± 4.76	7.45 ± 6.93	0.70	0.68	0.51	0.48
AC	−1.10 ± 0.84	13.4 ± 12.9	4.32 ± 6.91	6.60 ± 9.03	0.25	0.32	0.78	0.56
IC	−0.16 ± 0.64	11.3 ± 9.44	−3.39 ± 9.06	4.15 ± 2.13	0.77	0.29	0.49	0.12
Cg	−0.29 ± 1.45	7.77 ± 9.06	−3.97 ± 3.83	1.19 ± 5.16	0.81	0.60	0.33	0.82
mPFC	0.56 ± 1.10	7.17 ± 9.83	−0.14 ± 5.57	4.34 ± 6.28	0.61	0.72	0.83	0.69
Sep	−1.41 ± 1.17	11.4 ± 8.20	−7.57 ± 7.73	2.11 ± 4.94	0.31	0.44	0.28	0.75

MEMRI signal response (T_1_ and T_1_-w, means ± SEM) in each brain region was compared in the shCx43-LV-injected side versus the shCTRL-LV-injected side in each mouse (paired *t* test). Abbreviations: SC, Somatosensory cortex; Cpu, Caudate putamen; Dra, Dorsal raphe pallidus; Hip, Hippocampus; Hyp, Hypothalamus; LC, Locus coeruleus; LPB, Lateral parabrachial nucleus; MC, Motor cortex; OB, Olfactory bulb; SN, Substantia nigra; ThN, Thalamic nuclei; OC, Orbital cortex, PrL, Prelimbic cortex; AC, Auditory cortex; IC, Insular cortex; Cg, Cingulate cortex; mPFC, Medial prefrontal cortex; Sep, Septum.

Quantification of MEMRI signal responses (T_1_, T_1_-w) in 17 supplementary brain regions from mice injected with recombinant LV showed that no significant difference between the shCx43-LV injected side and the contralateral shCTRL-LV injected side was observed outside the hippocampus (Table 2). Thus, regardless the device on which the data were acquired: 7 T or 11.7 T, significant differences in the T_1_ relaxation time and the T_1_-weighted signal intensity were noted only at the hippocampal level (Table 2).

### Mefloquine treatment normalized interhemispheric differences in MEMRI signal responses in shCx43-LV injected mice

Mice with unilateral selective silencing of Cx43 expression in hippocampal astrocytes received MnCl_2_ i.p. injection two weeks after intra-hippocampal LV administration, and were treated 24 h later with MEF or vehicle i.p. 2.5 h before MEMRI signal acquisition (Fig. 1H). The ratio shCx43/shCTRL for T_1_-weighed intensity in the Cx43 knockdown hippocampus over that in the contralateral control hippocampus was compared to the theoretical value, i.e. 1, expected for equal values on both sides. In concordance with data obtained in the previous experimental series (Fig. 2G), the mean T_1_-weighted ratio was significantly different from 1 (n = 6; p < 0.05; one-sample *t* test) in unilateral Cx43 knockdown mice treated with the vehicle (Fig. 2I). In contrast, after MEF administration, no difference was found in hippocampal T1-weighted intensity between the ShCx43-LV-injected side and the contralateral shCTRL-LV injected side (n = 5; p = 0.562; ns; one-sample *t* test, Fig. 2I). Quantification of T_1_-weighted ratio of MEMRI signal intensity in Cx43-LV-injected side over that in the contralateral shCTRL-LV-injected side showed that none of the same 17 brain regions as those selected - in addition to the hippocampus - in the previous experimental series presented any significant difference in MEF- versus vehicle-treated mice (Table 3).

**Table 3:** Effects of mefloquine (MEF) treatment on MEMRI signal in various brain regions of mice with unilateral hippocampal Cx43 silencing.

Region	Ratio T_1_-w		Statistics	
	shCx43-LV/shCTRL-LV		shCx43-LV/shCTRL-LV	
	Veh	MEF	Veh	MEF
**Hip**	**1**.**15 ± 0**.**05**	**1**.**06 ± 0**.**10**	<**0**.**05**	**0**,**56**
SC	1.09 ± 0.07	1.04 ± 0.16	0.22	0.79
Cpu	1.10 ± 0.07	1.05 ± 0.16	0.20	0.75
DRa	1.09 ± 0.07	1.08 ± 0.19	0.28	0.71
Hyp	1.08 ± 0.08	1.09 ± 0.20	0.33	0.67
LC	1.11 ± 0.06	1.06 ± 0.13	0.15	0.67
LPB	1.14 ± 0.07	1.07 ± 0.12	0.11	0.58
MC	1.09 ± 0.07	1.03 ± 0.17	0.21	0.85
OB	1.10 ± 0.08	1.08 ± 0.18	0.29	0.68
SN	1.14 ± 0.07	1.07 ± 0.13	0.090	0.60
ThN	1.13 ± 0.07	1.07 ± 0.16	0.11	0.70
OC	1.10 ± 0.08	1.08 ± 0.19	0.26	0.71
PrL	1.08 ± 0.10	1.10 ± 0.24	0.48	0.70
AC	1.13 ± 0.13	0.97 ± 0.14	0.35	0.86
IC	1.11 ± 0.09	1.03 ± 0.16	0.29	0.88
Cg	1.08 ± 0.09	1.07 ± 0.21	0.43	0.74
mPFC	1.07 ± 0.10	1.08 ± 0.22	0.50	0.74
Sep	1.11 ± 0.08	1.09 ± 0.19	0.22	0.66

In both MEF- and vehicle (Veh)-treated mice, data are expressed as the ratio of MEMRI signal intensity in each brain area on the shCx43-LV-injected side over the shCTRL-LV-injected side. Statistical analyses were made by comparing each ratio (mean ± SEM) with the hypothetical value 1. Abbreviations: SC, Somatosensory cortex; Cpu, Caudate putamen; Dra, Dorsal raphe pallidus; Hip, Hippocampus; Hyp, Hypothalamus; LC, Locus coeruleus; LPB, Lateral parabrachial nucleus; MC, Motor cortex; OB, Olfactory bulb; SN, Substantia nigra; ThN, Thalamic nuclei; OC, Orbital cortex, PrL, Prelimbic cortex; AC, Auditory cortex; IC, Insular cortex; Cg, Cingulate cortex; mPFC, Medial prefrontal cortex; Sep, Septum.

### MEMRI signal responses to Cx43 blockers in mouse hippocampus

We then investigated the effect, *in vivo*, of three pharmacological blockers of Cx43, MEF, MFA and FLE on MEMRI signal in the hippocampus of intact healthy mice (without intra-hippocampal LV injections) that had received MnCl_2_ via the i.p. route 24 h before (Fig. 2A). Figure 2B showed the MEMRI signal intensity of selected coronal sections (both, −1.7 mm from bregma) of a representative vehicle-treated mouse (left panel) in comparison with a MEF-treated mouse (right panel). These images show a clear-cut increase in hippocampal MEMRI signal after MEF treatment. Quantification of T_1_-w signal intensity in 6 mice in each group demonstrated that MEF-induced increase was statistically significant (+18.0 ± 6.0%, p < 0.05). As shown in Fig. 2C, both FLE and MFA also significantly increased the T_1_-w signal intensity in the mouse hippocampus, up to the same level as that found with MEF (FLE: + 18.0 ± 4.7%, p < 0.05; MFA: + 18.0 ± 4.3%, p < 0.01).

## Discussion

Data from this study present a new application of MEMRI to investigate the functional activity of cerebral astroglial Cx43 *in vivo*, and to determine the effects of pharmacological blockers of astroglial Cxs in the mouse brain.

Neurons have long been considered as the primary performers of brain processes. However, during evolution, glia-to-neuron ratio significantly increases towards more and more adapted species. Among glial cells, astrocytes are closely connected to neurons and interactions between these two types of cells have largely been described^22^. Astrocytes have been successfully labeled with PET probes such as ^11^C-acetate^23^, ^11^C-DED and ^11^C-Sch225336^10^, passive fluorescent dyes such as sulforhodamine 101^11^ or genetically encoded Ca^2+^ indicators (GECIs)^12^. However, neurons, as well as astrocytes, readily take up blood-born lactate^24^, and so far, the use of GECIs or dyes has been limited due the requirement of their local intracerebral application and the low penetrance depth of optical microscopy^13^. *In vivo* brain imaging of the interactions between astrocytes and neurons is hence still challenging.

The functional activity of astrocyte networks involves key transmembrane proteins called Cxs^5^. Eleven Cx isoforms have been detected in the brain, and, among them, astrocytes express Cx43 and Cx30 isoforms in adult brains. While Cx30 is abundantly present in the thalamus, Cx43 and Cx30 are expressed at similar levels in the hippocampus^6,25^. The coupling between astrocytes is significantly reduced in cells lacking Cx43, demonstrating that this isoform is a major support for direct intercellular communication in astrocytes, notably in the hippocampus^6,26^. Additionally, Cx43-mediated networks have been shown to play an important role in several neuronal modulation processes^2,6–8,27^. In the present study, aimed at evaluating the role of Cx43 in Mn-based imaging, we silenced Cx43 expression in the hippocampus on one side and used the contralateral hippocampus as control. Unilateral Cx43 knockdown was achieved by intra-hippocampal injection of a lentiviral recombinant vector, shCx43-LV, pseudotyped with the MOKOLA envelop, and encoding a shRNA directed against Cx43 RNA within hippocampal astrocytes but not hippocampal stem cells or neurons^17,18^. Immunohistochemical controls fully confirmed that extinction of Cx43 expression occurred in astrocytes of the (randomly left or right) hippocampus injected with shCx43-LV. In contrast, Cx43 expression was preserved in the contralateral hippocampus injected with the control recombinant virus, shCTRL-LV, encoding non-relevant shRNA (shRNA against GFP). Interestingly, semi-quantitative estimates of DAPI labeling showed that none of these recombinant viruses had neurotoxic effects.

To compare the Mn-enhanced MR signal intensity in both sides of the hippocampus, as well as in other brain regions, LV-injected mice were administered MnCl_2_ i.p. 24 hours before imaging, i.e. under time conditions allowing Mn^2+^ concentration and distribution to reach equilibrium in brain^28^. The MnCl_2_ dose (50 mg/kg i.p.) was chosen to avoid any neurological symptoms or neurotoxicity^29^. MEMRI analyses on both hippocampal sides indicated that T_1_ was significantly shortened when Cx43 expression was inhibited. T_1_-weighted signal confirmed this observation within the hippocampus. In contrast, MEMRI analyses in the other 17 brain regions examined did not reveal any difference between the shCx43-LV injected side and the contralateral shCTRL-LV injected side. These data indicate that manganese was more concentrated only in the hippocampus where Cx43 expression was silenced^14,30^. There are two possible explanations for this differential manganese accumulation between the two hippocampi: (i) Mn^2+^ accumulates more in astrocytes on the shCx43-LV injected side as a result of a reduced diffusion through deficient gap junctions or decreased release caused by the lower density of Cx43 hemichannels or (ii) Mn^2+^ accumulates more in neurons as a functional consequence of Cx43 downregulation in astrocytes. As a matter of fact, although Mn^2+^ accumulation specifically in astrocytes, neurons, or other cells cannot be determined with the presently available methods, our study reveals that down regulation of Cx43 selectively in hippocampal astrocytes leads to changes in the local MEMRI signal and provides a first *in vivo* validation of a functional role for astrocyte Cx43 using whole brain imaging. Noteworthy, similar results were obtained with 7 and 11.7 Tesla fields, widening the potential applications of MEMRI using different types of equipment. Validation in other brain areas, with different expression levels of Cx43, such as cortex or striatum^31^, might also be interesting, especially regarding regional molecular heterogeneity of hemichannels and gap junctions involved in astrocyte-neuron interactions^32^. The respective contribution of hemichannels or gap junctions in the transport of manganese remains an important issue, which is, however, complex to evaluate due to the lack of specific *in vivo* tools. Interestingly, using a cell line derived from normal adult human osteoblasts, Romanello *et al*.^33^ highlighted that hemichannels participated in Mn^2+^ uptake from the external medium. On the other hand, Niessen *et al*.^34^ demonstrated that Mn^2+^ had the ability to diffuse within monolayers of transfected HeLa cells expressing murine Cx26, Cx32 as well as Cx43. These two series of data support the idea that both hemichannels and gap junctions are involved in manganese transport within adjacent cytoplasms and/or between cytoplasm and extracellular space.

Although *in vitro* studies on cultured astrocytes led Lu *et al*.^35^ to report that Cx43 expression could be affected by Mn^2+^, the cation concentration required for this effect, 125–1000 μM, was higher than that which was probably reached in brain under our *in vivo* conditions, i.e. 24 h after a unique injection of MnCl_2_ at 50 mg/kg. With such conditions, available literature data on brain accumulation of peripherally injected MnCl_2_^36,37^ led us to estimate that Mn^2+^ did not exceed 50 µM at the time of MEMRI signal acquisition. Even if direct measurements of brain Mn concentration will be needed to definitively exclude any possible alteration in Cx43 expression, it has to be emphasized that such alteration would occur equally in both hippocampi. Since our analyses consisted of comparing the hippocampus on the shCx43-LV-injected side with that on the contralateral shCTRL-LV-injected side, any global change in brain Cx43 caused by Mn^2+^ would not challenge the meaning of our data.

The next step for further investigations on the *in vivo* functional role of astrocyte Cx43 would be to quantify MEMRI signal in response to Cx43 up regulation specifically in astrocytes in the mouse hippocampus. However, to date, recombinant LV for Cx43 overexpression specifically in astrocytes are still under development and are not yet available for such studies.

It is well established that MEF inhibits Cx43-mediated functions, through gap junctions^38,39^ and hemichannels^40^, potentially via loop gating^41^. Interestingly, in naive healthy mice, MEF significantly increased the MEMRI signal in the hippocampus, supporting the idea that in Cx43 knock-down animals, MEF concealed the disequilibrium in MEMRI signal between both hippocampi by increasing the signal on the shCTRL-LV injected side up to that already achieved by Cx43 knockdown on the shCx43-LV injected side. Interestingly, the other two drugs tested, FLE and MFA, which are known to cross the brain-blood barrier and exert clear-cut inhibitory effects on Cx43 (but through still uncharacterized mechanisms)^42,43^, similarly increased MEMRI signal in the hippocampus in naive healthy mice, further supporting the idea that MEMRI signal in this brain region is related to Cx43 function.

Meanwhile, targeting Cxs, using in particular MEF, FLE and MFA, can result in changes in the properties of CNS drugs, in terms of pharmacological profiles^40,42^. Such techniques as MEMRI may help determining the mechanism of action of pharmacological combinations of CNS drugs with Cx targeted compounds, notably in terms of localization of Cx modulations in brain. Indeed, Cx expression displays regional heterogeneity within and between brain regions^9^. Furthermore, Cx functions can also be differentially regulated by local and region specific modulators such as neurotransmitters^9^. Interestingly, in our study, MEF restored equilibrium of the T_1_ relaxation time of MEMRI signal between both hippocampi in mice injected with shCx43-LV on one side and shCTRL-LV on the contralateral side, which provided a direct demonstration of an *in vivo* action of MEF on Cx43-mediated function in brain.

MEMRI has already been investigated in humans, notably with the use of FDA-approved mangafodipir, a Mn^2+^ chelate with the ligand fodipir^44^. Translational application of the present study, possibly with the use of mangafodipir, might provide new insights into neuron-astrocyte interactions in clinical trials and constitute a new tool for pharmacological evaluation of new modulators of those interactions. Additionally, as Cx43 expression is altered in neurodegenerative disorders such as Alzheimer’s disease^5,45^, Parkinson’s disease, Huntington’s disease, amyotrophic lateral sclerosis^46,47^ and multiple sclerosis^5,48^, as well as in psychiatric disorders such as depression^18,49^, Cx43 function evaluation using MEMRI might also be interesting for further pathophysiological investigations of those disorders. The diversity of Cx isoforms in the brains combined with their diverse functions should be further investigated to fully understand their roles in the brain.

### Significance statement

Astrocytes are key non-neuronal cells implicated in many brain functions. These glial cells are organized in functional networks, mostly through connexin 43 (Cx43)-mediated gap junction channels. Astroglial networks are engaged in fine neuronal tuning and have recently been considered as emerging new therapeutic targets in CNS disorders. However, comprehensive understanding of the complex relationships between astrocytes and neurons *in vivo* is limited due to the lack of adequate imaging tools. Here, we demonstrate that manganese enhanced magnetic resonance imaging (MEMRI) is a promising technique for studying *in vivo* the function of astrocyte Cx43 and assessing the pharmacological profiles of Cx43 modulators.
